# Identification and validation of oxidative stress-related immune signatures in Hepatitis B Virus associated acute liver failure based on WGCNA and machine learning

**DOI:** 10.1371/journal.pone.0356638

**Published:** 2026-09-09

**Authors:** Lu Dai, Jungang Tan, Zhimin Zhou, Chengcong Chen, Guoxin Hu, Ling Guo

**Affiliations:** 1 Department of Infectious Diseases, Peking University Shenzhen hospital, Shenzhen, China; 2 Clinical Institute of Anhui Medical University & Peking University Shenzhen hospital, Shenzhen, China; 3 Department of Radiation Oncology, Guangzhou Institute of Cancer Research, the Affiliated Cancer Hospital, Guangzhou Medical University, Guangzhou, China; National Taiwan University Hospital, TAIWAN

## Abstract

**Background:**

Acute liver failure (ALF) is a serious clinical disease. Immune infiltration and oxidative stress (OS) play an important role in ALF, but the combination of oxidative stress and immune infiltration in ALF has not been explored.

**Methods:**

Differential expression analysis and weighted gene co-expression network analysis (WGCNA) were performed on ALF and control samples in the GSE96851 dataset to obtain DE-OS-MGs. Candidate OS-IM-associated signature genes were mined using the least absolute shrinkage and selection operator (LASSO) and support vector machine (SVM) machine learning. Finally, OS-IM-related signature genes were obtained by expression analysis and ROC validation. Single gene set enrichment analysis (GSEA), TF-miRNA-mRNA network construction and drug prediction were also performed for OS-IM-related signature genes.

**Results:**

Differential analysis identified a total of 3580 DEGs in ALF and control samples, and differential analysis of immune cells yielded 15 immune cell species that were significantly different between the two groups. After WGCNA analysis, 1134 key module genes were screened. Then, 3580 DEGs, 855 OS-related genes, and 1134 key module genes were intersected, resulting in a total of 89 DE-OS-MGs. Furthermore, machine learning was performed and the genes were intersected to obtain 5 candidate OS-IM-related signature genes. The expression levels and ROC analysis of the 5 genes were validated in the GSE96851 and GSE120652 datasets, and 4 genes (CLU, APOH, AMBP, and GPX8) were used as biomarkers (OS-IM-related signature genes) for subsequent analysis. Single-gene GSEA enrichment analysis revealed that the 4 biomarkers were associated with metabolic processes. A miRNA-mRNA network with 123 nodes and 194 relationship pairs was obtained. 49 TFs of 3 biomarkers (APOH, AMBP, and GPX8) and 56 relationship pairs of TFs-mRNA regulatory network also constructed. Searching for therapeutic drugs of 4 biomarkers, a drug-mRNA network of CLU and 3 drugs was constructed.

**Conclusion:**

In this study, we combined immune infiltration landscape and oxidative stress based on bioinformatic analysis to screen for diagnostic markers (CLU, APOH, AMBP, and GPX8) of ALF. It provides a new idea and theoretical basis for the therapeutic diagnosis of ALF.

## 1. Introduction

Acute-on-chronic liver failure (ACLF) is a severe syndrome defined by acute hepatic decompensation in patients with chronic liver disease, frequently involving a cytokine storm, multiple organ failure, and high short-term mortality [1]. As a major form of liver failure globally, ACLF is especially prevalent in the Asia-Pacific region, where chronic hepatitis B virus (HBV) infection is the leading cause [2]. The pathophysiology of HBV-related ACLF (HBV-ACLF) is multifaceted, encompassing viral reactivation, a dysregulated immune response, and immunometabolic disturbances [3]. Accurately predicting clinical outcomes for HBV-ACLF patients remains difficult. Although prognostic scores like the Model for End-Stage Liver Disease (MELD) and MELD-Na are widely used to assess mortality risk in end-stage liver disease, they demonstrate considerable limitations for short-term prognosis in HBV-ACLF [4,5]. Consequently, discovering novel and reliable biomarkers is urgently required to improve early diagnosis and prognostic evaluation for these patients.

Emerging evidence implicates oxidative stress as a pivotal driver of ALF progression [6]. Hepatocellular integrity is maintained by the Nrf2-Keap1 signaling axis, which governs the transcriptional activation of cytoprotective antioxidant genes [7–11]. However, despite the established protective role of Nrf2 in liver pathology, the specific contribution of oxidative stress-related genes to HBV-ALF pathogenesis remains underinvestigated. Notably, the potential of these genes to serve as diagnostic biomarkers is yet to be explored.

Concurrently, immune dysregulation is central to ALF pathogenesis. Recent transcriptomic analyses have delineated the immune landscape of HBV-ALF, revealing upregulated apoptosis and necroptosis pathways [12]. Notably, ALF subtypes exhibit distinct immune signatures based on etiology: HBV-ALF is characterized by an enrichment of activated NK cells and plasma cells, diverging from the T-cell dominance observed in acetaminophen-induced ALF [13].The etiological heterogeneity of HBV-ACLF necessitates an investigation into its specific immune-oxidative stress interactions.

While oxidative stress and immune infiltration are independently recognized as critical determinants of liver failure, their potential synergistic interplay in HBV-ALF remains undefined. In this study, we integrated oxidative stress signatures with immune infiltration patterns using transcriptomic data from patients with HBV-related acute liver failure (HBV-ALF). This combined bioinformatics approach aimed to identify novel diagnostic biomarkers for the condition. The analysis also sought to provide new mechanistic insights into HBV-ALF.

## 2. Materials and methods

### 2.1 Data sources

ALF-related expression profile data were obtained from the GEO database (https://www.ncbi.nlm.nih.gov/geo/), including the GSE96851 dataset (tissue samples from 17 controls and 17 HBV-ALF patients) and the GSE120652 dataset (tissue samples from 3 controls and 3 HBV-ALF patients). 855 oxidative stress-related genes were obtained from GeneCards (https://www.genecards.org).

### 2.2 Differential expression analysis

First, background correction and normalized log-transformation by the RMA method were performed using the R package oligo (version 1.60.0), and duplicate genes were filtered according to expression quartile distance (IQR). Then, the samples in GSE96851 dataset were subjected to principal component analysis (PCA) [14]. And the R language limma package (version 3.46.0) [15] was used to compare the differential gene expression levels between the ALF and control groups (the screening condition was |log_2_FC| > 1 and adjusted p-value < 0.05). The volcano map was drawn using ggplot2 (version 3.3.6). And the expression trend of genes was demonstrated using pheatmap (version 1.0.12).

### 2.3 Immuno-infiltration analysis

CIBERSORT algorithm (version 1.03) [16] was used to calculate the proportion of 22 kinds of immune cells in ALF and control samples from GSE96851 dataset. Heat map and violin plot were plotted to visually compare the abundance of immune cells in the samples.

### 2.4 Weighted gene co-expression network analysis (WGCNA)

WGCNA (version 1.70.3) [17] was performed on the samples in GSE96851. A hierarchical clustering tree was first constructed for all samples (N = 34), sample dendrogram and trait (immune cells type) heat map were constructed. Next, gene modules were constructed by selecting appropriate soft thresholds based on near scale-free topology criteria. Modules were then collected according to the criteria of the dynamic tree-cutting algorithm. Correlations between each gene module and trait (immune cells type) were calculated, and the key module was selected. Finally, MM (Module membership) and GS (Gene significance) screening were performed, and the key module genes were screened using the criteria of |MM| > 0.8 and |GS| > 0.6, respectively.

### 2.5 Functional enrichment and PPI network construction

Intersections of DEGs, oxidative stress genes, and key module genes were taken using the R package venn (version 1.11) [18], which were recorded as DE-OS-MGs. Based on the expression patterns in ALF samples, the DE-OS-MGs were divided into up-regulated and down-regulated subgroups. GO function and KEGG pathway analysis for the two subgroups were performed separately using the clusterprofiler package (version 4.2.2) [19]. The STRING database was used to obtain protein interaction information, which was used to create PPI network for DE-OS-MGs.

### 2.6 LASSO and SVM-RFE algorithm model

The LASSO analysis [20] of the DE-OS-MGs was performed using glmnet (version 4.1.2) to obtain the cross-validated error map and gene coefficient map. The R package e1071 (version1.7–9) [21] was used to perform SVM analysis on the DE-OS-MGs, then the RFE was used to rank the importance of genes. The candidate OS-IM-related signature genes were uncovered by intersecting the genes obtained by LASSO and SVM-RFE.

### 2.7 Expression analysis and ROC validation

The expression levels of the candidate OS-IM-related signature genes were verified in the GSE96851 and GSE120652 datasets and the differences between the ALF and control groups were tested using t-test. ROC curves were drawn by pROC package (version 1.18.0) [22] to evaluate the diagnostic value of the candidate genes, with the area under the curve (AUC) greater than 0.7 indicated a diagnostic significance. The larger the AUC, the higher the accuracy. Finally, the OS-IM-related signature genes were obtained.

### 2.8 Model robustness validation

To further evaluate the stability and reliability of the diagnostic model constructed based on biomarkers, 10-fold cross-validation and Bootstrap resampling methods were employed for analysis. The 10-fold cross-validation used the trainControl function in the caret package to set the cross-validation parameters (using stratified sampling strategy, method = “cv”, number = 10), and enabled the calculation of class probabilities (classProbs = TRUE), using the area under the ROC curve (AUC) of the binary classification model as the evaluation metric. The logistic regression model was fitted using the train function, with biomarkers as the predictor variables, and the AUC results from 10 cross-validations were aggregated to calculate the average AUC, standard deviation, and 95% confidence interval. Bootstrap resampling used the boot function in the boot package for resampling with replacement, with 2000 iterations (R = 2000), and the sample size of each resampling was consistent with the original dataset. A custom AUC calculation function was defined to fit the logistic regression model for each resampled sample, and the roc and auc functions in the pROC package were used to calculate the AUC.

### 2.9 Gene Set Enrichment Analysis (GSEA)

Single-gene GSEA [23] analysis was performed to find the significant biological functions and pathways of the OS-IM-related signature genes. The filtering criteria were |NES| > 1, p. adjust < 0.05, and q. value < 0.2.

### 2.10 The TF-miRNA-mRNA network construction and drug prediction

The miRcode (http://mircode.org) database was used to screen the regulatory miRNAs associated with the OS-IM-related signature genes, and the miRNA-mRNA regulatory network was mapped using cytoscape (version 3.8.2) software. The ENCORI database was selected to predict the TFs of the signature genes and construct a regulatory network of TF-mRNA. The therapeutic drugs for the OS-IM-related signature genes were retrieved from the DGIDB database (https://dgidb.genome.wustl.edu/), and a drug-gene interaction network was constructed.

### 2.11 Animal experiments and APAP-induced liver injury model

Male C57BL/6 wild-type (WT) mice, aged 6–8 weeks and weighing 20–25 g, were used in this study. The animals were maintained in a specific pathogen-free (SPF), temperature-controlled facility (22 ± 2°C) under a 12-hour light/dark cycle, with free access to standard laboratory chow and water. All animal care and experimental procedures strictly followed the ARRIVE guidelines and were approved by the Institutional Laboratory Animal Care and Use Committee (IACUC) of Peking University Shenzhen Hospital (Approval No. 2024−160). An acute liver injury model was established by administering a single intraperitoneal (i.p.) injection of acetaminophen (APAP) at 300 mg/kg [24]. Control animals received an equivalent volume of normal saline (NS) via i.p. injection. To minimize distress, all potentially painful procedures were performed under isoflurane inhalation anesthesia. After APAP administration, the mice were monitored every 12 hours for health and behavioral changes. Pre-defined humane endpoints were applied to prevent unnecessary suffering; any animal showing severe distress, including profound lethargy, a hunched posture, ruffled fur, or unresponsiveness to stimuli, was scheduled for immediate euthanasia. At the designated time points (Day 1 and Day 2), the mice were euthanized via isoflurane overdose. Following confirmation of death, serum and liver tissue samples were collected for subsequent analysis.

### 2.12 Biochemical analysis

Serum samples were separated from whole blood by centrifugation. The levels of serum alanine aminotransferase (ALT) were detected using commercial kits according to the manufacturer’s instructions to evaluate the extent of liver injury [24].

### 2.13 Histological analysis

Liver tissues were harvested and fixed in 4% paraformaldehyde for 24 hours. The tissues were subsequently dehydrated, embedded in paraffin, and cut into 4-μm-thick sections. The sections were deparaffinized, rehydrated, and stained with hematoxylin and eosin (H&E) for morphological observation under a light microscope.

### 2.14 RNA extraction and quantitative real-time PCR (RT-qPCR)

Total RNA was isolated using the EZ-press RNA Purification Kit (EZBioscience, Cat. B0004DP). The concentration and purity of RNA were determined using a NanoDrop spectrophotometer. Complementary DNA (cDNA) was synthesized using a Reverse Transcription Kit. Quantitative real-time PCR (RT-qPCR) was performed using SYBR Green Master Mix on a real-time PCR system. The relative mRNA expression levels of target genes (AMBP, CLU, APOH, GPX8, and GSTP1) were calculated using the 2^–ΔΔCt method, with GAPDH serving as the internal control. The primers used for amplification were as follows: GAPDH forward, 5’-ACAACTTTGGTATCGTGGAAGG-3’ and reverse, 5’-GCCATCACGCCACAGTTTC-3’; CLU forward, 5’-GCTGCTGATCTGGGACAATG-3’ and reverse, 5’-ACCTACTCCCTTGAGTGGACA-3’; APOH forward, 5’-GCCATGATCGGAAATGACACA-3’ and reverse, 5’-GCCGTCCAGCTTGTATGTCTC-3’;AMBP forward, 5’-GTGGTCCACACCAACTATGACG-3’ and reverse, 5’-GCCACATCCTTGAACTCCTGCA-3’; GPX8 forward, 5’-TGCGAGACAGAACTACGGAGTC-3’ and reverse, 5’-TTCCACCTTGGCTCCTTCTTGG-3’; GSTP1 forward, 5’-TGGAAGGAGGAGGTGGTTACCA-3’ and reverse, 5’-GGTAAAGGGTGAGGTCTCCATC-3’.

### 2.15 Statistical analysis

All bioinformatics analyses were undertaken in R language. The rank sum test and t-test were employed to contrast the data from different groups.

### 2.16 Ethical approval

The study protocol followed the ethical principles of the 1975 Declaration of Helsinki and was approved by the Ethical Committee and Institutional Review Boards of Peking University Shenzhen hospital.

## 3. Results

### 3.1 Screening of DEGs in ALF

**Fig 1:**
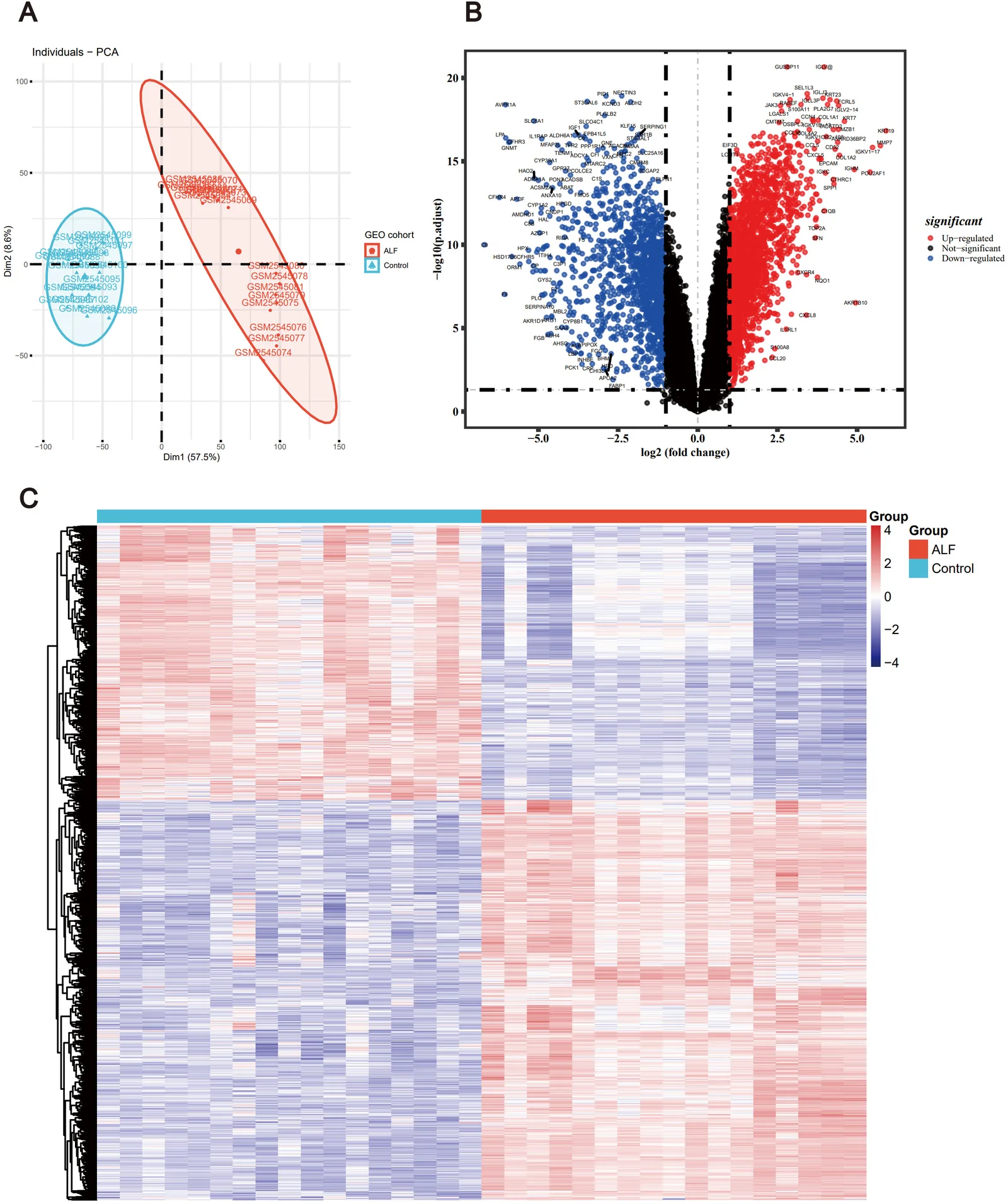
Identification of differentially expressed genes (DEGs) in hepatitis B virus-associated acute liver failure (HBV-ALF). (A) Principal Component Analysis (PCA) showing the distinct distribution between HBV-ALF samples and normal control samples in the GSE96851 dataset. (B) Volcano plot displaying the distribution of DEGs. Red dots represent upregulated genes (n = 2123), and blue dots represent downregulated genes (n = 1457) based on the criteria of |log_2_FC| > 1 and adjusted P < 0.05. (C) Hierarchical clustering heat map illustrating the expression patterns of the top DEGs across the HBV-ALF (pink bar) and control (blue bar) groups. The color scale from blue to red indicates low to high gene expression levels.

First, background correction and normalized log-transformation were conducted by the RMA method, and duplicate genes were filtered according to IQR. The stability of the different samples was checked by PCA, and the results showed fine quality of the data (Fig 1A). Differential analysis obtained a total of 3580 DEGs in ALF samples, of which 2123 genes were up-regulated and 1457 genes were down-regulated in ALF samples (Fig 1B). The expression of up-regulated and down-regulated genes were presented in the heat map (Fig 1C).

### 3.2 Identification of differential immune cells in ALF

**Fig 2:**
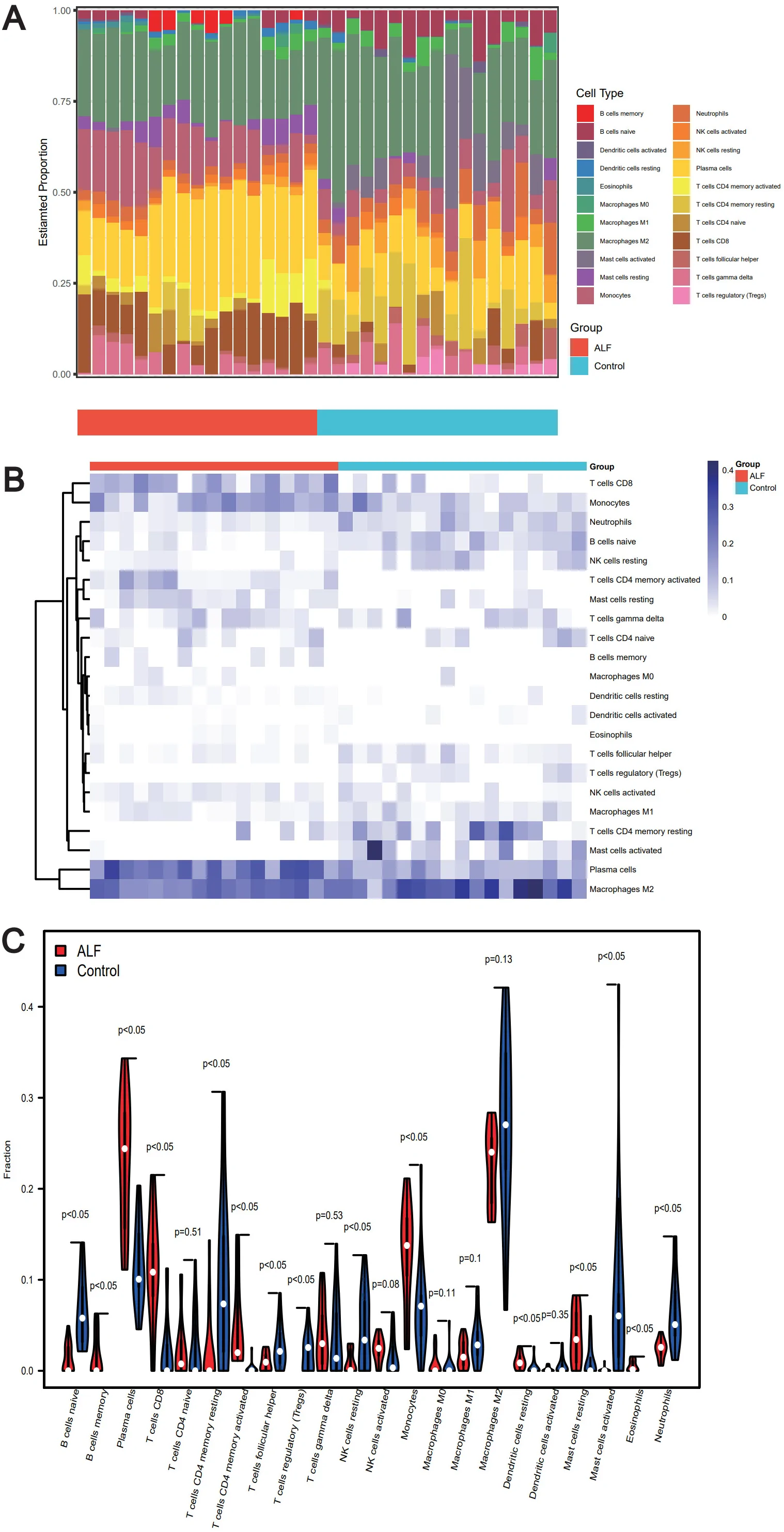
Landscape of immune cell infiltration in the ALF microenvironment. (A) Stacked bar chart visualizing the relative proportions of 22 infiltrating immune cell types in each sample from the GSE96851 cohort, estimated using the CIBERSORT algorithm. (B) Heatmap showing the abundance of immune cell populations across individual samples. (C) Violin plots comparing the infiltration levels of 22 immune cell types between the ALF (red) and control (blue) groups. Statistically significant differences (P < 0.05) were observed in 15 cell types, including Plasma cells, Macrophages, and Neutrophils.

By analyzing the ratio of 22 immune cell species in the HBV-ALF and control groups, bar graph of the proportion of immune cells in each sample and heat map of the abundance of immune cells in the sample were constructed to demonstrate the immune cell content between the HBV-ALF and control groups (Fig 2A and 2B). In the HBV-ALF group and the control group, there were significant differences in the quantities of 15 types of immune cells (naive B-cell, memory B-cell, plasma cells, CD8 + T cell, resting CD4 + memory T cell, activated CD4 + memory T cell, follicular helper T cell, regulatory T cell (Tregs), resting NK cells, monocytes, resting dendritic cells, resting mast cells, activated mast cells, eosinophils, and neutrophils) (Fig 2C).

### 3.3 Mining of differential immune cell-associated module genes

**Fig 3:**
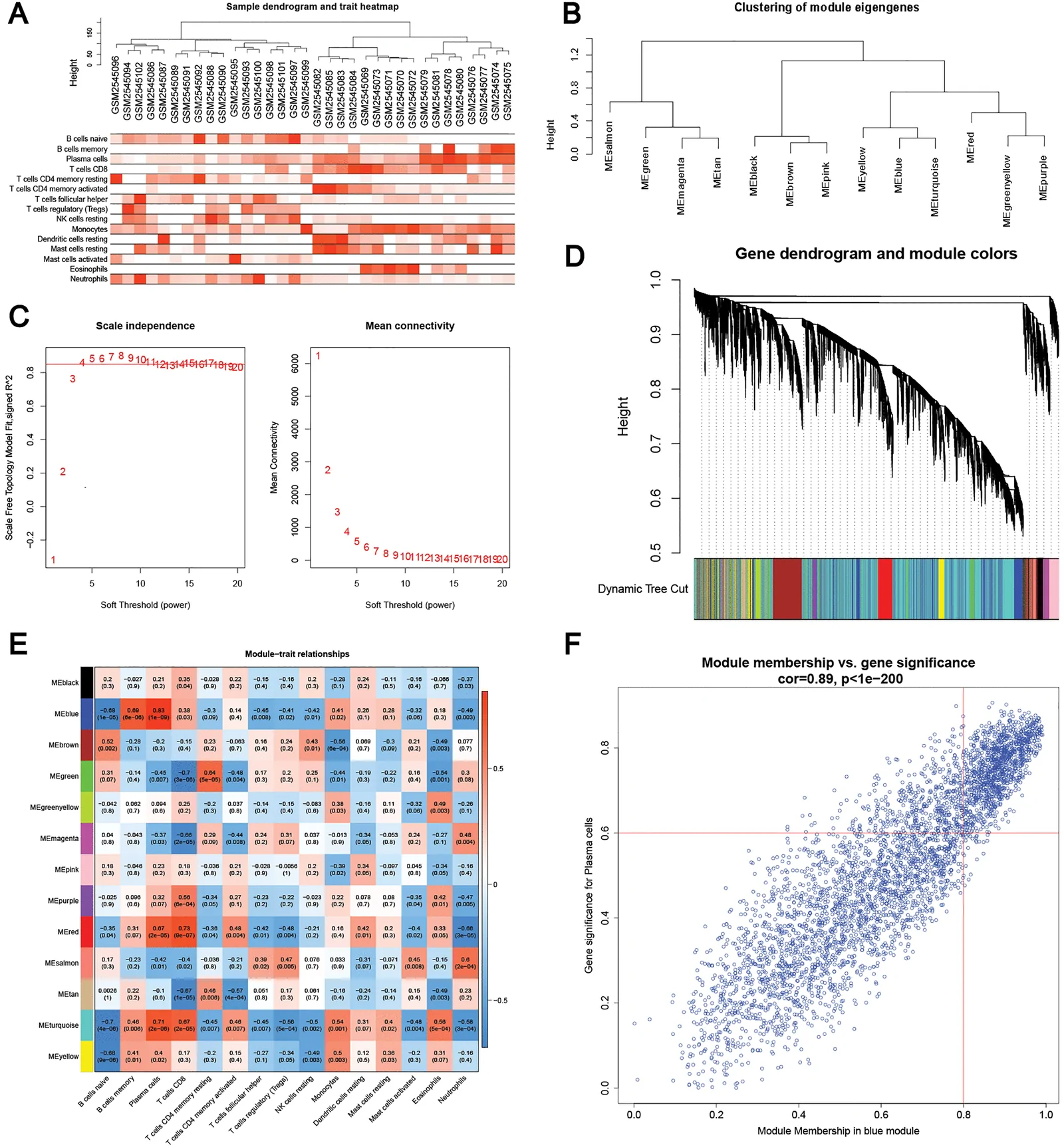
Construction of a Weighted Gene Co-expression Network (WGCNA) to identify immune-related modules. (A) Sample dendrogram and trait heatmap linking gene expression clusters to clinical traits (immune cell types). (B) Clustering dendrogram of module eigengenes. (C) Determination of the soft-thresholding power (β = 4) to ensure a scale-free network topology. Left: Scale-free fit index; Right: Mean connectivity. (D) Gene dendrogram obtained by hierarchical clustering, with colored bands representing identified co-expression modules. (E) Module-trait relationship heatmap. The “blue” module showed the highest positive correlation with Plasma cells (r = 0.83, P < 1e-200). (F) Scatter plot of Module Membership (MM) vs. Gene Significance (GS) for Plasma cells in the blue module. Genes with |MM| > 0.8 and |GS| > 0.6 were identified as key module genes.

WGCNA analysis was performed to find module genes associated with the differential immune cells. The sample clustering result indicated that there were no outliers, as shown in the sample dendrogram and trait (immune cell type) heat map (Fig 3A). 4 was chosen as the best soft threshold to construct gene modules (Fig 3B), and finally 14 gene modules were obtained, among which the gray module was non-sense (Fig 3C and 3D). Among all module-immune cell relationship pairs, the blue module and plasma cells had the highest correlation (cor = 0.83, p < 0.01). So we selected the blue module as the key module, in which 1134 genes that met the criteria of |MM| > 0.8 and |GS| > 0.6 were identified as immune-related key module genes (Fig 3E and 3F).

### 3.4 Enrichment analysis and PPI network construction of DE-OS-MGs

**Fig 4:**
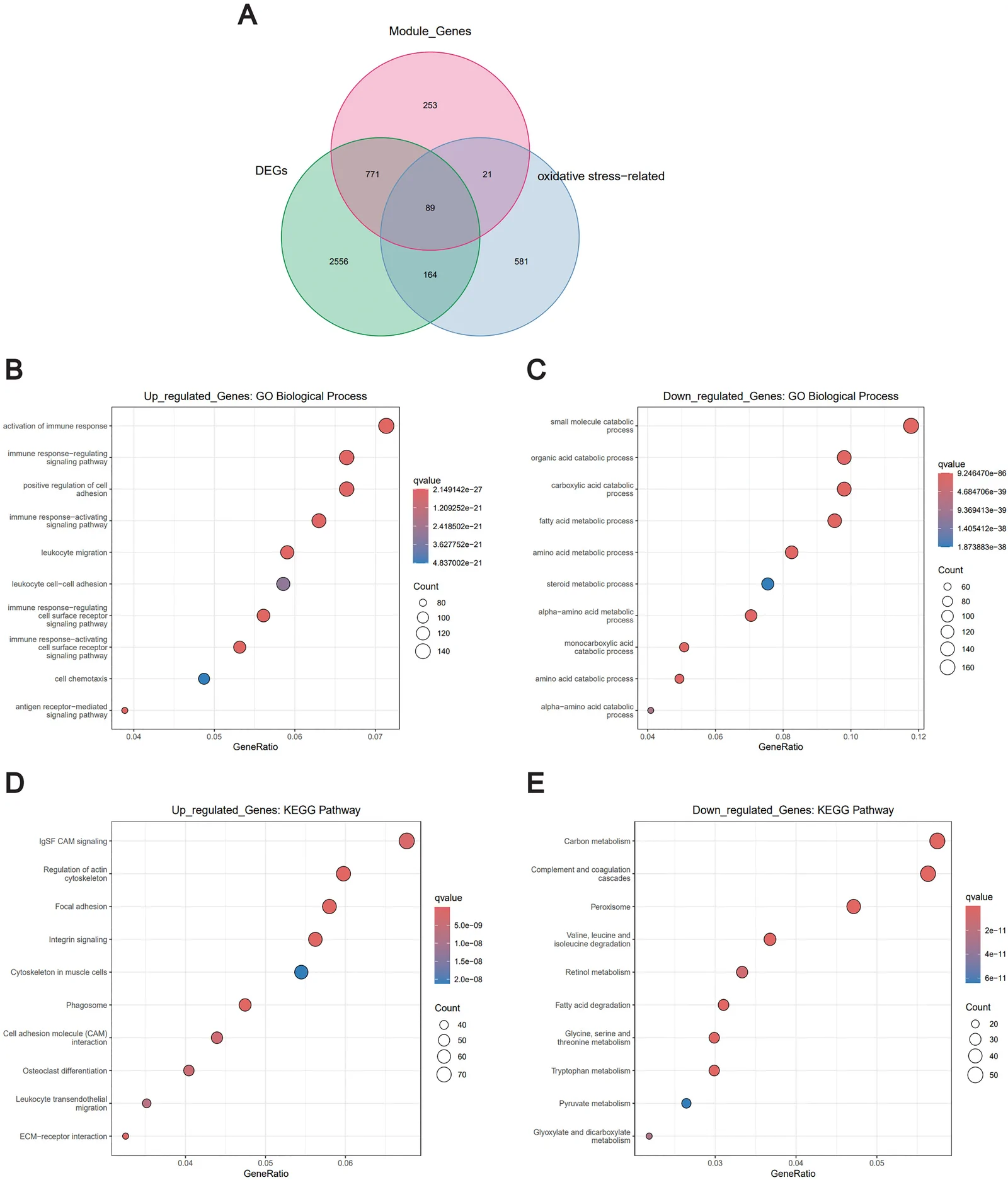
Functional enrichment and interaction network analysis of Oxidative Stress-Immune-Related Module Genes (DE-OS-MGs). (A) Venn diagram illustrating the intersection of DEGs, oxidative stress-related genes (OSGs), and key WGCNA module genes, resulting in 89 overlapping DE-OS-MGs. (B) Upregulation of GO pathway enrichment analysis for DE-OS-MGs. (C) Downregulation of GO pathway enrichment analysis for DE-OS-MGs. (D) Upregulation of KEGG pathway enrichment analysis for DE-OS-MGs. (E) Downregulation of KEGG pathway enrichment analysis for DE-OS-MGs.

Intersections of DEGs, oxidative stress genes, and key module genes were taken, resulting in 89 DE-OS-MGs (Fig 4A). GO and KEGG enrichment analysis was performed for DE-OS-MGs. A total of 562 GO processes were enriched (p.adjust < 0.05 and count > 2), including 445 BP items, 39 CC items, and 78 MF items (S1 Table). GO and KEGG enrichment analyses were conducted separately for the upregulated and downregulated DE-OS-MGs. The GO terms enriched by the upregulated genes mainly involved immune response activation, leukocyte migration, and positive regulation of cell adhesion, etc. (Fig 4B). The GO terms enriched by the downregulated genes mainly involved small molecule decomposition metabolism, organic acid/carboxylic acid decomposition metabolism, fatty acid and amino acid metabolism, etc. (Fig 4C). The KEGG pathway enrichment analysis results showed that the upregulated genes were mainly enriched in IgSF CAM signaling, focal adhesion, integrin signaling, and leukocyte transendothelial migration, etc. (Fig 4D), while the downregulated genes were significantly enriched in metabolic-related pathways, including carbon metabolism, complement and coagulation cascade reaction, peroxisome, fatty acid degradation, etc. (Fig 4E). Fig 4D and 4E present the top enriched KEGG pathways for up- and down-regulated genes, while the complete list of all significantly enriched KEGG pathways is provided in S2 Table. In order to investigate the linkage between DE-OS-MGs, predictions were made using the STRING website, and a protein-protein interaction network (PPI) was constructed with 88 nodes and 619 edges (Fig 4F).

### 3.5 Mining for OS-IM-related signature genes

**Fig 5:**
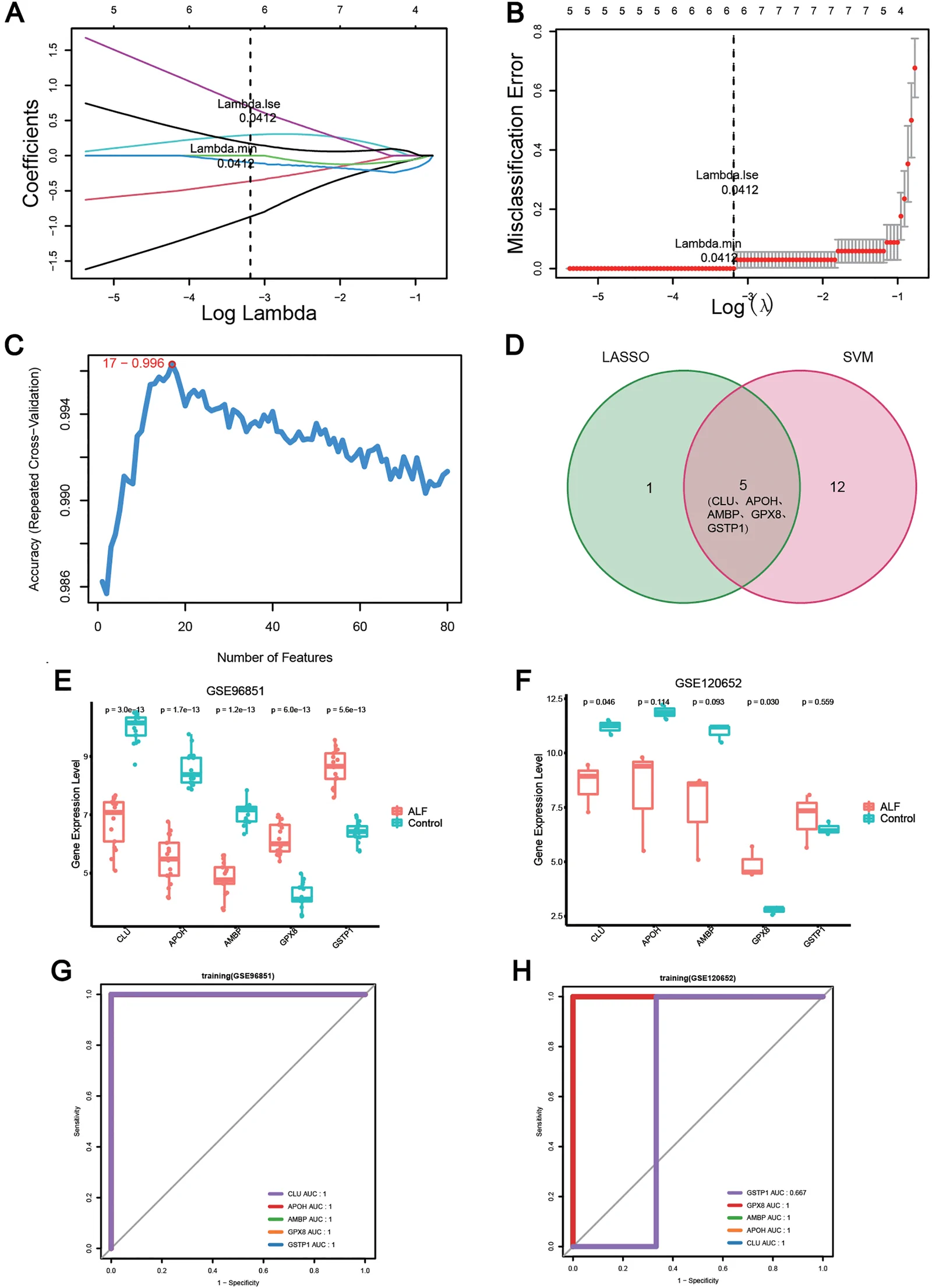
Screening and validation of diagnostic biomarkers using machine learning algorithms. (A, B) LASSO regression analysis. (A) Coefficient profiles of the features. (B) Selection of the optimal parameter using 10-fold cross-validation. (C) Support Vector Machine-Recursive Feature Elimination (SVM-RFE) algorithm for feature selection, identifying a subset of genes with the highest accuracy. (D) Venn diagram showing the 5 candidate biomarkers (CLU, APOH, AMBP, GPX8, GSTP1) identified by the intersection of LASSO and SVM-RFE models. (E, F) Boxplots validating the expression levels of the candidate genes in the training set (GSE96851, E) and the external validation set (GSE120652, F). (G, H) Receiver Operating Characteristic (ROC) curves evaluating the diagnostic performance of the biomarkers in the training (G) and validation (H) datasets. Four genes (CLU, APOH, AMBP, GPX8) exhibited high diagnostic accuracy (AUC > 0.7).

The LASSO analysis screened 6 signature genes (CLU, APOH, AMBP, CRYAB, GPX8, and GSTP1) from the DE-OS-MGs (Fig 5A and 5B). Meanwhile, SVM-RFE screening based on the DE-OS-MGs obtained 17 signature genes (Table 1) (Fig 5C). Then, the genes obtained by Lasso analysis and SVM-RFE algorithms were intersected to obtain 5 candidate OS-related signature genes (CLU, APOH, AMBP, GPX8, and GSTP1) (Fig 5D). The expression of the candidate OS-related signature genes in the ALF and control samples of the GSE96851 was analyzed (Fig 5E), in which GPX8 and GSTP1 were highly expressed, and CLU, APOH, and AMBP were lowly expressed in ALF group. The expression trend of the genes in GSE120652 dataset was completely consistent with GSE96851 dataset (Fig 5F). In addition, in the GSE96851 and GSE120652 datasets, the AUC values of CLU, APOH, AMBP, and GPX8 were greater than 0.7, indicating that they could distinguish ALF patients from control samples and had potential diagnostic value (Fig 5G and 5H). Therefore, these 4 genes were authenticated as OS-IM-related signature genes (or biomarkers) for subsequent analysis. However, their true diagnostic value still needs to be further validated in larger, multicenter, independent clinical cohorts.

**Table 1:** SVM-RFE screening based on the DE-OS-MGs obtained 17 signature genes.

Feature Name	Feature ID	Average Rank
CP	1	4.8
AMBP	45	6.6
GPX8	85	7.2
AOX1	9	7.4
GSTP1	87	7.4
F2	8	7.9
APOH	25	8.2
SLC25A4	71	8.6
AR	22	9.7
GCH1	58	12.3
MAOA	41	13.7
PPARA	44	15.3
CFTR	89	15.8
KNG1	5	16.3
CLU	24	16.4
HP	26	17.9
SERPINA1	31	19.2

Notes: Feature Name, gene symbol of the candidate feature; Feature ID, numeric identifier of the feature in the dataset; Average Rank, mean importance rank across cross-validation folds.

### 3.6 Model robustness validation results

The 10-fold cross-validation results showed that the average AUC of the model was 1, with a 95% confidence interval of [1, 1] (S1A Fig). The Bootstrap resampling results indicated that the median AUC of the model was 1, and the 95% confidence interval was [1, 1] (S1B Fig). These results suggest that this diagnostic model exhibits excellent discrimination ability under different data subset divisions, is not affected by random sampling fluctuations, and has good stability and statistical robustness.

### 3.7 Experimental validation of signature genes in APAP-induced ALF mouse model

**Fig 6:**
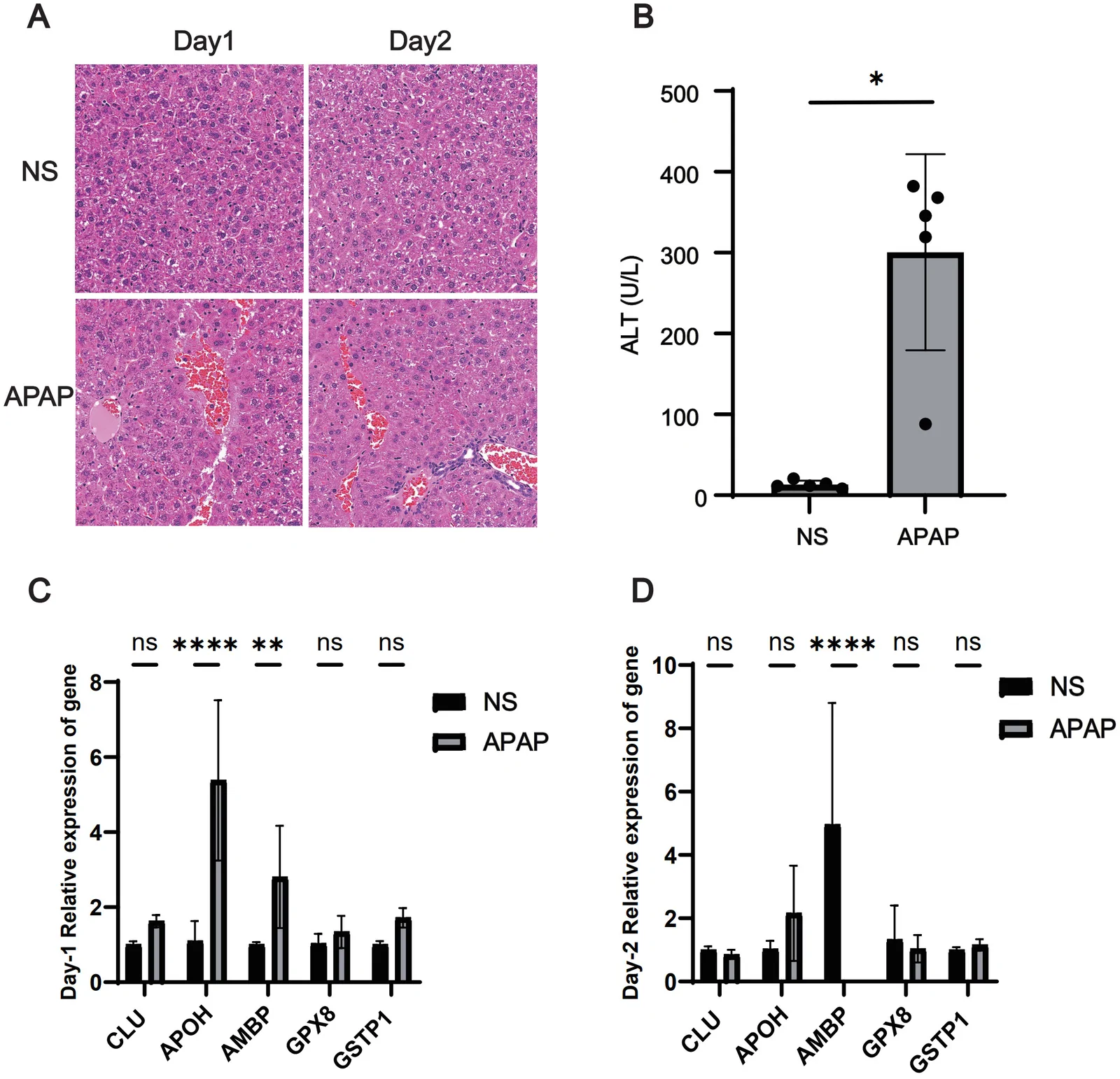
In vivo validation of signature genes in an APAP-induced acute liver injury mouse model. (A) Representative H&E staining images of liver sections from the Control (NaCl) and APAP-treated groups at Day 1 and Day 2 (Magnification: 40×). Note the necrosis and inflammatory infiltration in the APAP group. (B) Serum ALT levels indicating liver injury in the APAP group compared to controls. (C, D) RT-qPCR analysis of the relative mRNA expression of CLU, APOH, AMBP, GPX8, and GSTP1 in liver tissues at Day 1 (C) and Day 2 (D) post-injury. Data are presented as mean ± SD. P < 0.05, P < 0.01, P < 0.0001, ns: not significant.

The biological relevance of the identified signature genes was validated using an APAP-induced ALF mouse model. Successful modeling was confirmed by H&E staining, which displayed hepatocyte necrosis and inflammatory infiltration in the APAP group, contrasting with the intact architecture of saline controls (Fig 6A). This was corroborated by significantly elevated serum ALT levels (Fig 6B). RT-qPCR analysis revealed distinct temporal expression patterns for the signature genes (Fig 6D). On day 1 following APAP treatment, the expression levels of both APOH and AMBP were significantly higher than those in the control group, while there were no statistically significant differences in the expression levels of CLU, GPX8, and GSTP1. Based on the changes in APOH and AMBP expression (significantly elevated on day 1),. By day 2, APOH expression had returned to baseline levels (no difference from the control group), while AMBP expression was significantly lower than that of the control group. These in vivo data confirm the dysregulation of these oxidative stress markers during ALF, supporting our bioinformatics predictions.

### 3.8 Single-gene GSEA of OS-IM-related signature genes

**Fig 7:**
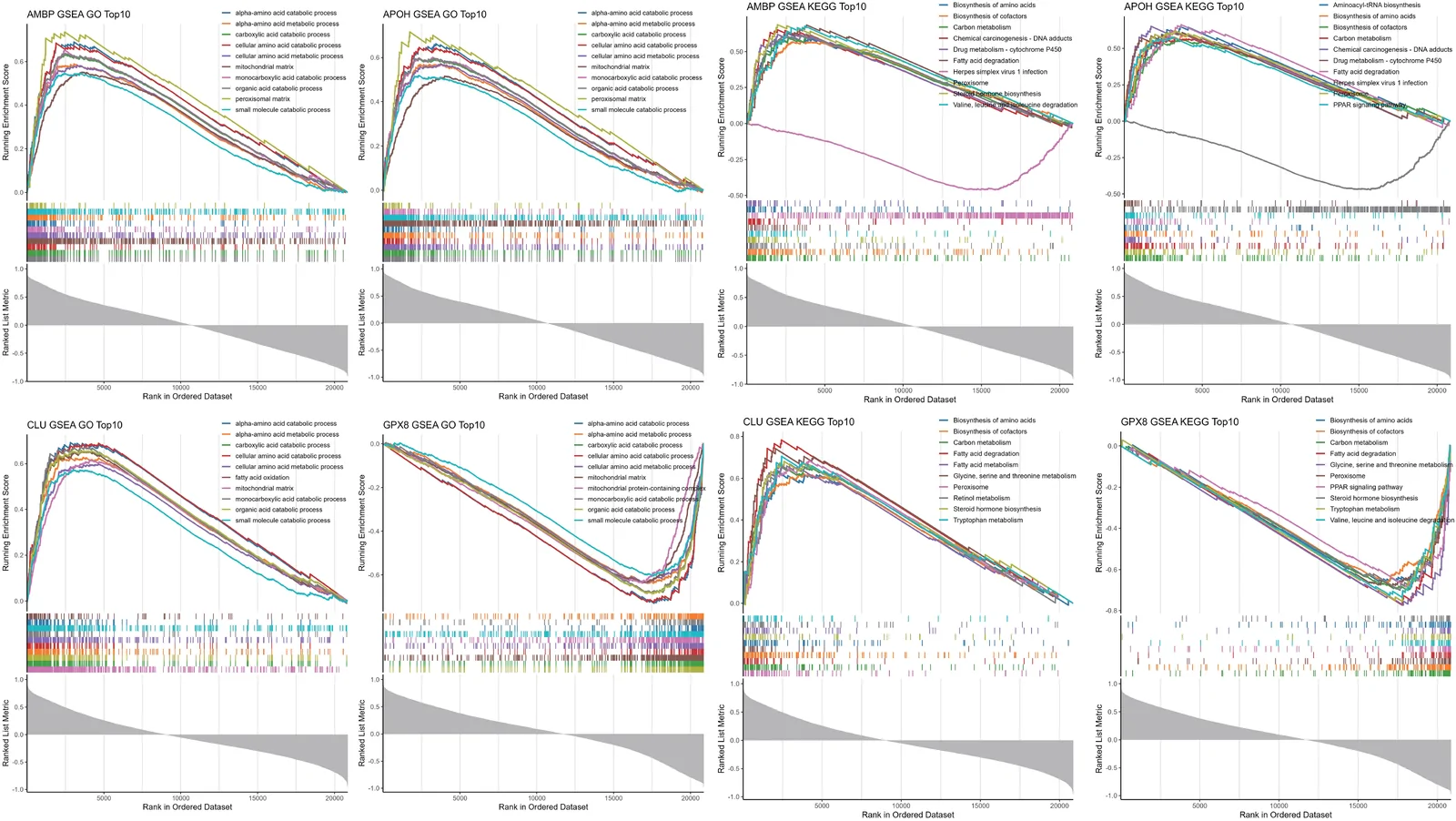
Single-Gene Set Enrichment Analysis (GSEA) of the identified biomarkers. (A) Top 10 Gene Ontology (GO) terms enriched for the four signature genes (AMBP, APOH, CLU, GPX8), primarily involving carboxylic acid catabolic processes and mitochondrial matrix functions. (B) Top 10 KEGG pathways enriched for the signature genes, emphasizing metabolic alterations such as fatty acid degradation and amino acid metabolism.

In order to understand the biological function and the involved signaling pathways of the OS-IM-related signature genes, GSEA was performed. The top 10 GO entries and KEGG pathways enriched by the 4 genes were displayed in (Fig 7A and 7B). We found that these genes were involved in carbon metabolism, the biosynthesis of cofactors, the peroxisome, organic acid catabolic processes, carboxylic acid catabolic processes, amino acid metabolism of cells, degradation of fatty acids, and the mitochondrial matrix etc. biological processes and pathways (Fig 7A and 7B).

### 3.9 Construction of TF-mRNA, miRNA-mRNA, and gene-drug networks based on OS-IM-related signature genes

**Fig 8:**
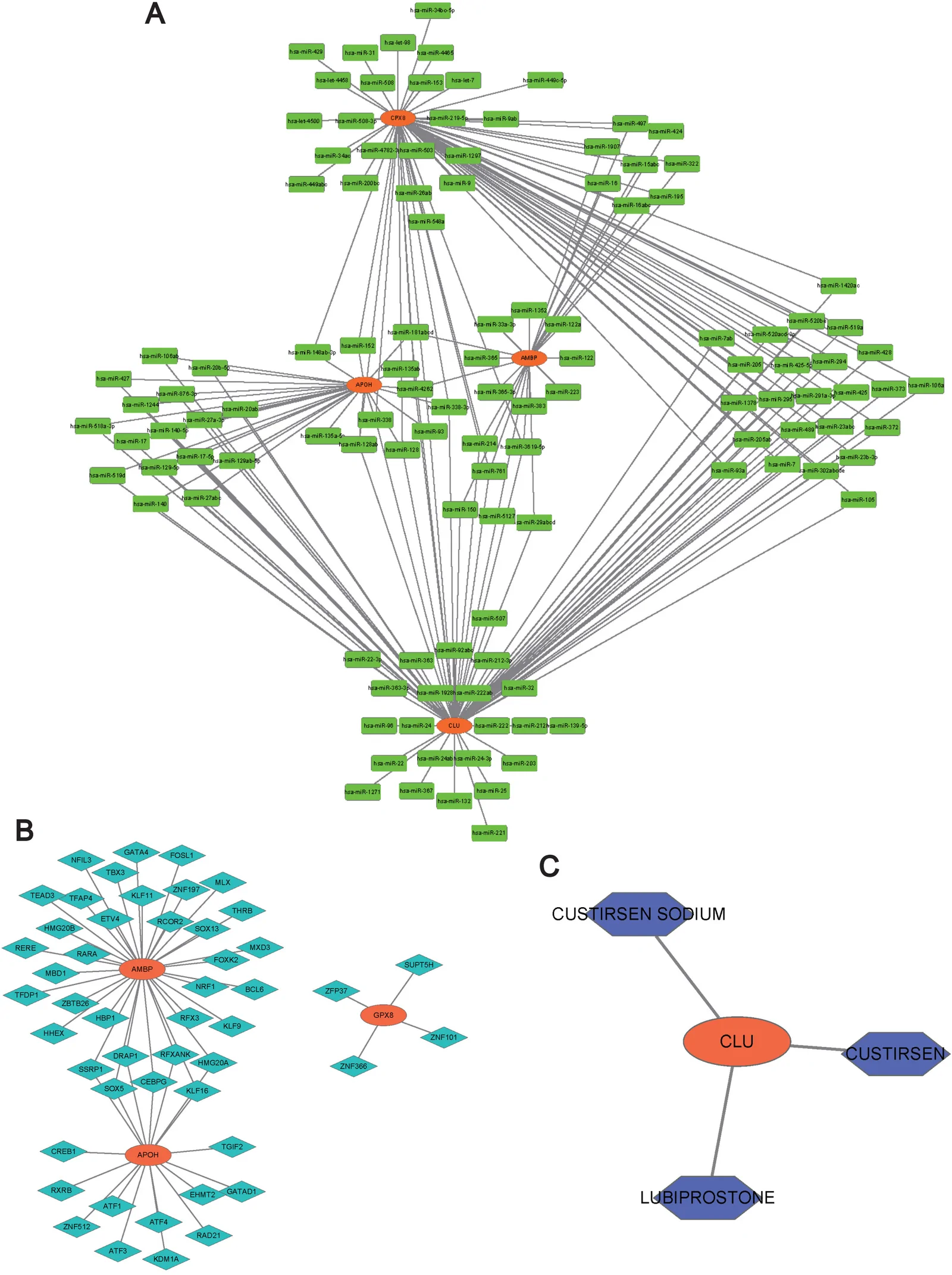
Construction of upstream regulatory and downstream therapeutic networks. (A) The miRNA-mRNA regulatory network illustrating potential interactions between the 4 biomarkers and 119 predicted miRNAs. (B) The Transcription Factor (TF)-mRNA regulatory network displaying interactions between biomarkers and 49 predicted TFs. (C) Drug-Gene interaction network identifying potential therapeutic agents (Custirsen, Lubiprostone) targeting CLU. Orange nodes represent the target gene, and purple hexagons represent candidate drugs.

The biomarker-related regulatory miRNAs were screened, including 24 miRNAs predicted by AMBP, 27 miRNAs predicted by APOH, 75 miRNAs predicted by CLU, and 66 miRNAs predicted by GPX8. After collation, 123 nodes (119 miRNAs and 4 biomarkers), and 194 relationship pairs were obtained to map the miRNA-mRNA regulatory network (Fig 8A). In addition, the TFs of 4 biomarkers were predicted, among which AMBP predicted 9 TFs, APOH predicted 13 TFs, and GPX8 predicted 10 TFs. Finally, after removing duplicates, 49 TFs were predicted for the 3 biomarkers. Based on the relationship pairs of TFs and mRNAs, TF-mRNA network containing 52 nodes and 56 relationship pairs was obtained (Fig 8B). In order to explore potential therapeutic drugs for biomarker-related diseases, we performed targeted drug identification. Based on the screened drugs, a drug-gene interaction network of biomarkers was constructed, which contained CLU and 3 drugs (CUSTIRSEN SODIUM, CUSTIRSEN, and LUBIPROSTONE) (Fig 8C).

## 4. Discussion

The rapid progression and high mortality of HBV-associated acute liver failure (HBV-ALF) necessitate a deeper understanding of the molecular crosstalk driving hepatic decompensation [25]. While the individual roles of immune dysregulation and oxidative stress are recognized [26], their synergistic impact on ALF pathogenesis has remained elusive. In this study, we bridged this gap by integrating transcriptomic profiling from the GSE96851 and GSE120652 datasets. By employing a multi-omics approach combining WGCNA, immune deconvolution, and machine learning, we identified a robust four-gene (CLU, APOH, GPX8, AMBP) oxidative stress signature inextricably linked to plasma cell infiltration, offering novel insights for diagnosis and therapeutic intervention.

Clusterin (CLU) is a multifunctional stress-inducible glycoprotein involved in apoptosis, lipid transport, membrane protection, and immune regulation [27,28]. Circulating CLU is primarily secreted by human hepatocytes, representing its main source [29]. Severe liver injury can therefore directly impair CLU production. Hepatocyte apoptosis and necrosis are central pathological events in the initiation and progression of liver failure. The development of acute-on-chronic liver failure (ACLF) frequently involves complex host immune dysregulation, where aberrant adaptive and innate immune responses significantly mediate hepatitis and hepatocyte apoptosis [30–32]. CLU protects cells from apoptosis induced by diverse stressors [33], indicating a potentially important protective function in liver injury. Several clinical studies support this perspective. For instance, CLU levels are significantly lower in patients with HBV-ACLF than in non-ACLF patients [34], a finding highly consistent with the markedly reduced CLU expression observed in the acute liver failure (ALF) group here. Given the associations identified in this study between CLU and metabolic pathways—such as fatty acid degradation and amino acid metabolism—as well as immune cell infiltration including plasma cells, the decline of CLU in HBV-ALF may signify a collapse of hepatoprotective mechanisms. This collapse would reduce tolerance to oxidative stress and immune-mediated damage, thereby accelerating the progression of liver failure.

Apolipoprotein H (APOH), or β-2-glycoprotein I, is a hepatocyte-synthesized glycoprotein primarily involved in lipid metabolism and cholesterol transport [35]. It docks with specific lipoproteins during triglyceride catabolism, activates lipoprotein lipase, and regulates both cholesterol transport and reverse cholesterol transport [35]. APOH has also been identified as a protective factor in acute liver injury [36]. In this study, APOH expression was low in patients with acute liver failure (ALF) and showed a dynamic pattern of initial elevation followed by suppression in a mouse model. This trajectory suggests APOH is activated on day 1 after APAP injection in liver injury to mitigate oxidative stress but becomes on day 2, resulting in a loss of protection. This dynamic change may offer biomarker utility for monitoring ALF progression.

Glutathione peroxidase 8 (GPX8) is a key member of the glutathione peroxidase family [37]. Localized to the endoplasmic reticulum (ER), it participates in metabolizing hydrogen peroxide, responding to ER stress, regulating apoptosis, and modulating calcium homeostasis and signaling [38]. GPX8 is therefore essential for maintaining cellular redox balance and protecting against oxidative damage [39]. Research on GPX8 has centered largely on cancer, where its functions display tissue specificity. In hepatocellular carcinoma, GPX8 acts as a tumor suppressor by inhibiting the Hsc70/AKT pathway [40]. By contrast, elevated GPX8 expression correlates with tumor progression in gastric cancer [41], breast cancer [42], and esophageal squamous cell carcinoma [43]. Although functional studies of GPX8 in the liver remain limited, its established role as an ER stress regulator implies it may also be important in liver injury. Here, we observed that GPX8 was significantly overexpressed in patients with acute liver failure (ALF), a finding consistently replicated across two independent datasets.

The α-1-microglobulin/bikunin precursor (AMBP) gene encodes a glycoprotein that is proteolytically cleaved to yield α-1-microglobulin (A1M) and bikunin, which regulate inflammation and inhibit proteases, respectively [44]. In this study, AMBP was downregulated in patients with acute liver failure (ALF) and displayed a dynamic pattern of initial elevation followed by suppression in a mouse model. PCR analysis further confirmed that the prognostic gene GSTP1 was highly expressed in the disease group, aligning with our bioinformatics findings.

Glutathione S-transferase P1 (GSTP1), a key member of the glutathione S-transferase family, catalyzes glutathione conjugation to detoxify xenobiotics [45,46]. This enzyme is crucial for regulating cellular oxidative stress and proliferation and serves as a significant serum biomarker in heart failure [47]. By catalyzing glutathione conjugation, GSTP1 protects cells from carcinogens and cytotoxic agents [48]. In HBV-related liver diseases, GSTP1 expression is epigenetically regulated [49]. For instance, hypermethylation of the GSTP1 promoter in HBV-related hepatocellular carcinoma significantly downregulates its transcription. Furthermore, during acute liver injury, GSTP1 exerts a protective effect by inhibiting the mitochondrial apoptosis pathway [50]. Our RT-qPCR analysis confirmed high GSTP1 expression in the disease group, consistent with the bioinformatics results and underscoring its stress-responsive role in liver injury. During machine learning screening, both LASSO and SVM-RFE algorithms retained GSTP1 as a candidate gene, indicating its potential to distinguish ALF from normal samples. However, subsequent ROC validation showed that the AUC values for GSTP1 in both the development and validation sets fell below the preset threshold of 0.7, so it was not included in the final diagnostic model. This outcome does not diminish the importance of GSTP1 in the pathophysiology of HBV-ALF but highlights a distinction between diagnostic biomarker screening and mechanistic studies: GSTP1 expression may show considerable inter-individual heterogeneity in ALF or be influenced by epigenetic factors like promoter methylation, reducing its stability as a standalone diagnostic indicator. Nevertheless, the central role of GSTP1 in the antioxidant defense network remains significant, and its functional study can provide important insights into the pathophysiology of HBV-ALF.

A pivotal finding of our study is the specific enrichment of plasma cells in the ALF immune microenvironment. Through CIBERSORT analysis and WGCNA, we identified plasma cells as the primary clinical trait correlated with the key “blue” gene module. This challenges the conventional T-cell-centric view of HBV pathogenesis and suggests that in the acute exacerbation phase, the humoral immune response may be hyperactivated. The massive infiltration of plasma cells likely represents a desperate host attempt to clear the viral load, yet this response may inadvertently fuel the cytokine storm and oxidative burst that destroy the hepatic parenchyma.

To distill a precise diagnostic signature from this immune-metabolic landscape, we applied a rigorous screening framework. Intersecting the module genes with oxidative stress markers and DEGs yielded 89 candidates, which were further refined using a dual machine learning approach (LASSO and SVM-RFE). This strategy minimized overfitting and identified five potential biomarkers. Notably, external validation in the GSE120652 cohort led to the exclusion of GSTP1 due to suboptimal discrimination (AUC < 0.7), resulting in a final panel of four high-confidence biomarkers (CLU, APOH, AMBP, GPX8). Functional enrichment and single-gene GSEA confirmed that these genes are deeply involved in lipid and amino acid metabolism, reinforcing the concept that metabolic collapse is a hallmark of ALF. First, GO and KEGG enrichment analyses revealed predominant enrichment in metabolic pathways such as peroxisome activity, fatty acid degradation, and carboxylic acid catabolism. This pattern contrasts with the findings of Zuo et al., who, using the same dataset, identified ferroptosis/autophagy-related hub genes primarily enriched in amino acid metabolism and immune-inflammatory responses [51]. The discrepancy suggests that the four genes identified here may represent a distinct “metabolism-oxidative stress” regulatory axis separate from ferroptosis, offering a novel framework for subtyping the molecular heterogeneity of HBV-ALF. Second, KEGG analysis also identified pathways involving tryptophan metabolism and monooxygenase activity. Given the strong correlation between the blue module from WGCNA and plasma cells (cor = 0.83, p < 0.01), these four genes may influence B cell differentiation into plasma cells and their function by modulating tryptophan metabolism and redox balance. This connection potentially links the otherwise separate processes of oxidative stress and humoral immune activation. Finally, single-gene GSEA indicated that the four genes were primarily enriched in amino acid catabolism pathways. The integrated enrichment analyses thus reveal a “metabolism-oxidative stress” axis distinct from ferroptosis and clarify a functional mechanism linking oxidative stress-related genes to plasma cell infiltration.

The biological relevance of these biomarkers is further illuminated by our regulatory network analysis. The construction of the miRNA-mRNA and TF-mRNA networks (involving 119 miRNAs and 49 TFs) suggests that the downregulation of metabolic genes and upregulation of stress responders are under tight, albeit dysregulated, transcriptional control. From a translational perspective, our drug-gene interaction scan identified CLU (Clusterin) as a promising therapeutic target, with three potential pharmacological agents identified. As a stress-induced chaperone, CLU may play a protective role in mitigating proteotoxicity during liver failure, making it an attractive candidate for drug repurposing.

Four marker genes exhibited significant differential expression in human HBV-ALF tissue samples (GSE96851), with CLU, APOH, and AMBP downregulated and GPX8 upregulated. In the APAP-induced mouse model of acute liver injury, however, APOH and AMBP were significantly upregulated on day 1 following APAP injection, a trend opposite to that seen in human samples. This apparent discrepancy can be explained by considering etiology, disease stage, and dynamic molecular changes. Human HBV-ALF results from persistent viral infection, involving long-term host gene regulation by viral proteins and immunopathological processes [52], whereas the APAP model represents an acute chemical insult lacking these specific viral-immunological features. Consequently, transcriptomic signatures differ substantially between these etiologies. The human samples were collected during the exhaustion phase following liver failure establishment, when protective genes are downregulated after sustained injury. The mouse model captures day 1 and day 2 after APAP injection. APOH and AMBP exhibited a rise-then-suppression dynamic expression was induced from day 1 to day 2, which suggests that the day 1 and day 2 following APAP administration may represent distinct stages. Finally, inherent species differences exist between mice and humans in liver metabolism, immune function, and oxidative stress regulation. Thus, the differing expression trends do not represent a contradiction but instead reveal the heterogeneity between HBV-ALF and APAP-ALI across etiology, pathological stage, and species, deepening our understanding of dynamic molecular events during ALF progression.

This study advances prior research on HBV-ALF in three key respects. First, it establishes an analytical framework centered on an “immune-oxidative stress interaction network,” integrating these two axes systematically, whereas Zuo et al. focused on ferroptosis/autophagy and Pei et al. on lactylation modification [51,52]. We identified four biomarkers—CLU, APOH, AMBP, and GPX8—of which CLU, APOH, and AMBP have not been systematically reported in HBV-ALF. Although GPX8 was noted by Ye et al. [53], our work is the first to incorporate it into a combined diagnostic model. Second, regarding immune characteristics, plasma cells emerged as the core immune feature (cor = 0.83, p < 0.01), contrasting with the reported dominance of CD4 + T cells by Pei et al. [52] and clarifying the critical role of humoral immunity in the HBV-ALF immune microenvironment. Finally, by validating the central role of oxidative stress pathways, the study provides novel insights across research perspective, biomarker combination, and immune characterization.

This study is the first to integrate immune infiltration and oxidative stress analyses, identifying biomarkers (CLU, APOH, AMBP, GPX8) closely associated with plasma cells in HBV-ACLF. Several limitations must be acknowledged. First, the transcriptomic analysis relied primarily on the GSE96851 dataset from patients with HBV-ALF, so the identified biomarkers may reflect molecular characteristics specific to that condition. Second, significant differences exist between the immune pathology of the acetaminophen (APAP)-induced mouse model of liver injury and that of clinical HBV-ALF. In the present study, however, disease progression was staged solely based on temporal changes in APOH and AMBP expression. The absence of dynamic quantitative comparisons, including hepatic necrosis area and inflammatory cytokine levels, constrains accurate assessment of disease severity at different time points in the model mice. Third, the work was based on bioinformatic predictions and expression validation, leaving the specific molecular regulatory mechanisms of the four-gene signature unexplored. Finally, although this study initially assessed the diagnostic value of four genes using ROC analysis, their actual diagnostic performance was not validated in an independent, large-scale clinical cohort. Furthermore, the relatively small size of the current dataset may increase the risk of overfitting. To address these limitations, future studies should incorporate multi-etiology, multicenter cohorts for external validation to assess biomarker generalizability. In animal models, moreover, dynamic changes in ALT levels, quantitative scores of hepatic necrosis area, and protective gene expression profiles at multiple time points should be integrated to more precisely distinguish the acute injury phase from the recovery phase. HBV-related transgenic or more clinically relevant ALF animal models are needed to validate the pathophysiological functions of the key genes. Techniques such as gene manipulation and single-cell sequencing could help elucidate the specific molecular mechanisms and regulatory networks involved. Prospective clinical studies, integrated with MELD/MELD-Na scores, should evaluate the incremental value of this signature for the early diagnosis and risk stratification of HBV-ALF to facilitate its clinical translation.

## 5. Conclusion

In conclusion, our study utilized comprehensive bioinformatics to delineate a specific plasma cell-associated oxidative stress signature in HBV-ALF. We successfully established a four-gene diagnostic model and mapped its upstream regulatory and downstream therapeutic networks. These findings provide a novel theoretical basis for understanding the immunometabolic axis in ALF and may offer potential targets for future biomarker-guided diagnosis and therapeutic exploration.

## Supporting information

S1 Fig The robustness verification results of the HBV-ALF diagnostic model. (A) 10-fold cross-validation performance. The box plot shows the distribution of AUC values from 10 iterations, with the blue dotted line representing the average AUC, and the average AUC value and 95% confidence interval are labeled. (B) Bootstrap resampling (2000 times) histogram of AUC distribution. The orange solid line represents the median AUC, the orange dotted line represents the 95% confidence interval, and the median AUC and 95% confidence interval are labeled.
(PDF)

S1 Table Gene Ontology (GO) enrichment analysis of differentially expressed genes in HBV-associated acute liver failure (HBV-ALF). Significantly enriched GO terms are categorized into Biological Process (BP), Cellular Component (CC), and Molecular Function (MF). Columns: ONTOLOGY, GO category; ID, GO term ID; Description, GO term name; GeneRatio, ratio of input genes annotated to the term; BgRatio, ratio of background genes annotated to the term; pvalue, hypergeometric test p-value; p.adjust, Benjamini-Hochberg adjusted p-value (FDR); geneID, input gene symbols mapped to the term; Count, number of input genes annotated to the term.
(CSV)

S2 Table KEGG pathway enrichment analysis of differentially expressed genes in HBV-associated acute liver failure (HBV-ALF). Columns: ID, KEGG pathway ID; Description, pathway name; GeneRatio, ratio of input genes annotated to the pathway; BgRatio, ratio of background genes annotated to the pathway; pvalue, hypergeometric test p-value; p.adjust, Benjamini-Hochberg adjusted p-value (FDR); geneID, input gene symbols mapped to the pathway; Count, number of input genes annotated to the pathway.
(CSV)
