# Supplementary material for: Identification and validation of oxidative stress-related immune signatures in Hepatitis B Virus associated acute liver failure based on WGCNA and machine learning
Source: PLoS One. 2026 Sep 9;21(9):e0356638. doi: 10.1371/journal.pone.0356638 (PMC13557375; doi:10.1371/journal.pone.0356638)
Supplement: Supplementary file 1 [file pone.0356638.s001.pdf]

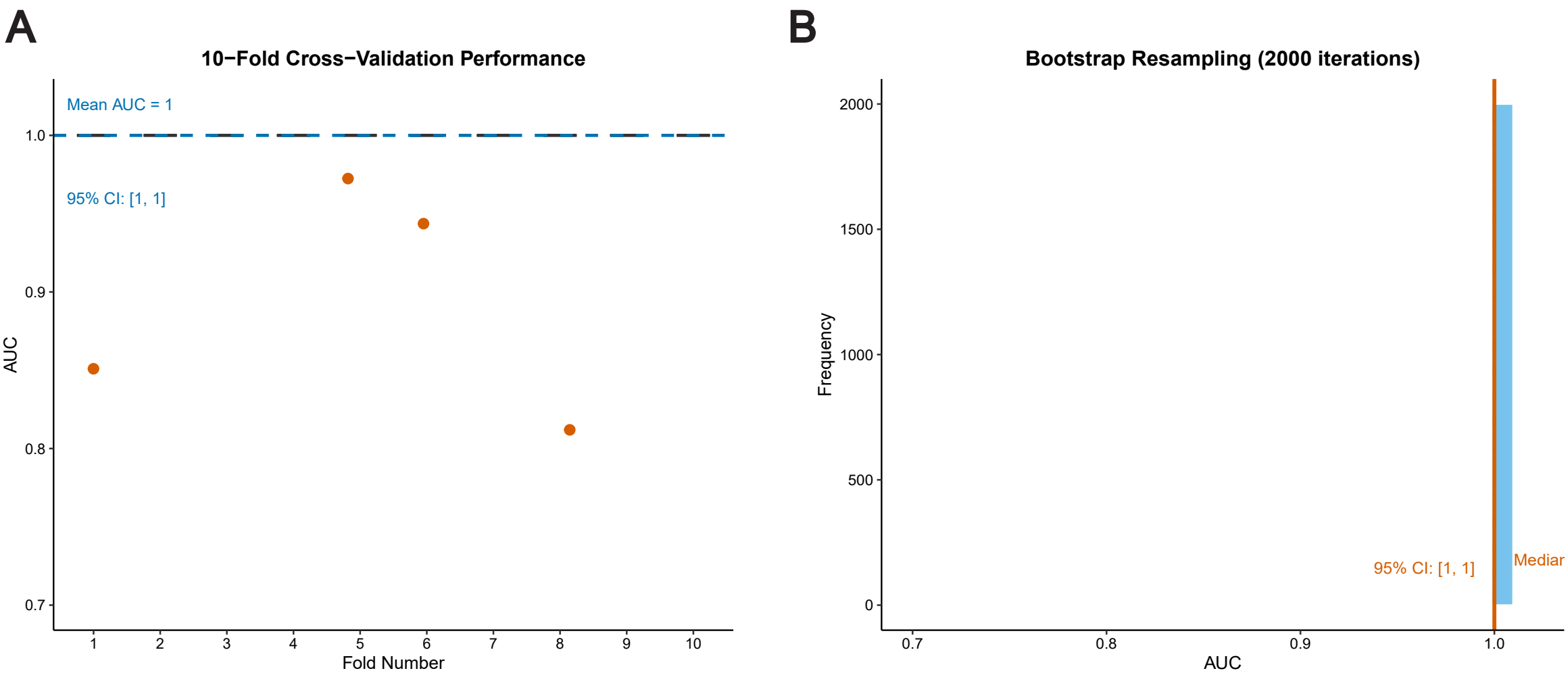
A
10−Fold Cross−Validation Performance
Mean AUC = 1
95% CI: [1, 1]
AUC
1.0
0.9
0.8
0.7
1
2
3
4
5
6
7
8
9
10
Fold Number
B
Bootstrap Resampling (2000 iterations)
Frequency
2000
1500
1000
500
0
95% CI: [1, 1]
Mediar
0.7
0.8
0.9
1.0
AUC
